# ‘No one's ever said anything about sleep’: A qualitative investigation into mothers' experiences of sleep in children with epilepsy

**DOI:** 10.1111/hex.13694

**Published:** 2023-01-06

**Authors:** Georgia Cook, Paul Gringras, Harriet Hiscock, Deb K. Pal, Luci Wiggs

**Affiliations:** ^1^ Department of Psychology, Health and Professional Development, Centre for Psychological Research, Faculty of Health and Life Sciences Oxford Brookes University Oxford UK; ^2^ Children's Sleep Medicine Evelina London Children's Hospital London UK; ^3^ Women and Children's Institute Kings College London London UK; ^4^ Health Services Research Unit Royal Children's Hospital Melbourne Victoria Australia; ^5^ Centre for Community Child Health Murdoch Children's Research Institute Melbourne Victoria Australia; ^6^ Department of Paediatrics The University of Melbourne Melbourne Victoria Australia; ^7^ Department of Basic and Clinical Neuroscience, Institute of Psychiatry, Psychology and Neuroscience King's College London London UK; ^8^ Medical Research Council Centre for Neurodevelopmental Disorders King's College London London UK; ^9^ Department of Paediatric Neuroscience King's College Hospital London UK

**Keywords:** child, epilepsy, experience, qualitative, sleep, support

## Abstract

**Introduction:**

Sleep problems in children with epilepsy (CWE) are common. However, little is known about parental experiences and feelings about managing sleep in their CWE. To provide the most appropriate services' provision, it is essential that the lived experience of parents of this patient group and the issues and problems that they face in managing their child's sleep is understood.

**Method:**

In 2018, nine mothers of CWE (aged 5–15 years) were interviewed about their perceptions and experiences around their child's sleep, sleep problems and their management, the impact of sleep difficulties on the child and their family and available support.

**Results:**

Four themes were identified that represented the nature of the child's sleep problems, including settling and night‐waking issues, parasomnias and child anxiety around sleep. Seven themes represented mothers' experiences of managing their child's sleep and any associated problems, including the longstanding challenging nature of child sleep issues, management strategies adopted, challenges related to managing sleep over time, the link between sleep and seizures, the negative impact of poor sleep on daytime functioning, role of antiseizure medication and maternal concerns about child sleep. One theme represented the perceived lack of information, help and support available.

**Conclusions:**

Findings suggest there are unmet needs in supporting parents to deal with sleep, sleep problems and their management in CWE.

**Patient or Public Contribution:**

This individual study was conducted under the umbrella of the CASTLE research programme (see https://castlestudy.org.uk/). Parents who have lived experience of parenting a child with epilepsy were co‐applicants for the programme and were involved in the original conception, aims, design and funding application for the research programme (including the project reported in this paper) and advised on project design. Mothers of CWE who have lived experience of managing sleep and sleep problems in their child were participants who shared their experiences through the interviews, which formed the data of the current study.

## 1 INTRODUCTION

Epilepsy is a common chronic neurological condition, characterized by recurrent seizures; the prevalence in children and adolescents under 18 years of age is estimated to be 0.32%–0.55%.^1^, ^2^ Many children with epilepsy (CWE) experience co‐existing deficits in behavioural, cognitive, attention, academic and psychosocial domains,^3^, ^4^, ^5^ as well as reduced quality of life in comparison to children without epilepsy.^6^, ^7^

Appropriate quantity and quality of sleep are crucial to healthy well‐being. However, sleep and epilepsy have a complex and bidirectional relationship^8^ and have been described as ‘unfortunate bedfellows’.^9^ In CWE, commonly reported sleep difficulties are problems around initiation (settling and falling asleep), maintenance (experiencing night or early morning wakings), duration, daytime sleepiness as well as sleep anxiety.^10^, ^11^, ^12^

Research into sleep deprivation of parents of children with complex needs has found that one of the biggest challenges was the need to be available or vigilant during the night and that the impact of this sleep deprivation is ‘relentless’ and ‘draining’.^13^ Over two‐thirds (68.6%) of parents in a previous study of parents of CWE reported having concerns about their child having night‐time seizures.^14^ These worries and concerns are likely to contribute to parental sleep disturbance, which is more common in parents of CWE than in parents of children without epilepsy.^15^ In a sample of parents of CWE (with intractable epilepsy), 75% reported that their sleep was affected, and of these, 48% reported the impact on their sleep was stressful.^16^

Traditional treatment goals for CWE tend to focus on seizure management, even though the importance of the need to develop child epilepsy care beyond ‘seizure control with minimal adverse effects’ has been a longstanding recommendation.^17^ Yet sleep or problems around sleep are not usually an aspect of standard care that is regularly addressed, even though sleep is a key consideration reported by parents, CWE and healthcare professionals.^18^, ^19^

Behavioural and educational approaches have shown signs of being effective treatments for many of the sleep difficulties experienced by these children and their families.^20^ Research suggests that there may be special considerations for managing sleep in CWE, which could helpfully be addressed to likely increase families' engagement with and feasibility of intervention.^21^ A greater understanding of these considerations could helpfully direct future intervention development^22^ and ensure broader service provision is focused on the areas of most need. However, little research has explored the lived experience of sleep and sleep problems in CWE on parents.

The current paper aimed to address the research questions: 1) explore the types of parentally reported sleep problems faced by CWE and their families, 2) identify parents' experiences and feelings around managing their child's sleep and any associated problems and 3) identify parents' perception of available help and support when parenting a CWE around sleep.

## 2 MATERIALS AND METHOD

This descriptive qualitative study was conducted as part of the CASTLE (Changing Agendas on Sleep, Treatment and Learning in Epilepsy) programme of studies. Parents were asked about their child's sleep, any sleep problems, the experience of parenting a CWE around sleep and their perception of available support as part of a wider interview to inform the development of an online sleep intervention.^21^, ^22^

### 2.1 Participants and recruitment

Participants were nine co‐habiting mothers of CWE (six boys and three girls who ranged in age from 5 to 15 years, with median = 10 years and mean = 10.3 years, SD = 2.9). See Table 1 for descriptive details about the children of the interviewees, including their epilepsy.

**Table 1:** Descriptive details about the children with epilepsy of interviewees

	Age	Gender	Type of epilepsy	Duration since diagnosis	Seizure timing
P1	10	M	Benign rolandic	<1 year ago	Transitioning between sleep and wake
P2	15	M	Benign rolandic (a‐typical)	>3 years ago	Daytime and during sleep
P3	10	F	Benign rolandic	<1 year ago	Transitioning between sleep and wake
P4	11	M	Benign rolandic	Between 1 and 3 years ago	Transitioning between sleep and wake
P5	5	F	Benign rolandic	Between 1 and 3 years ago	Daytime. 1 year without seizures (due to medication)
P6	9	M	Focal	>3 years ago	During sleep
P7	13	F	Unspecified	>3 years ago	During sleep and transitioning between sleep and wake
P8	7	M	Focal	>3 years ago	Transitioning between sleep and wake
P9	13	M	Generalized	>3 years ago	Daytime and transitioning between sleep and wake

Children suffered from a wide range of sleep problems, which allowed mothers to share their experiences of a broad range of sleep‐related issues, see Table 2 for a summary of the children's maternal‐reported sleep problems.

**Table 2:** Type of current and past parent‐reported sleep problems^a^

	Settling	Night waking	Early morning waking	Sleep‐related anxiety	Poor sleep quality	Daytime sleepiness	Morning waking difficulty	Co‐sleeping	Room sharing	Sleep terrors	Sleep walking	Other
P1 (child aged 10 years)	C	C		C	C			C		P	P	
P2 (child aged 15 years)	C	C	C	C		C			P	C	C	
P3 (child aged 10 years)		C		C			P	C		C		
P4 (child aged 11 years)	C	C		C					P			
P5 (child aged 5 years)	C	C		C		C		C	C			
P6 (child aged 9 years)	C	C	C			P		P				
P7 (child aged 13 years)	C						C					
P8 (child aged 7 years)		C	C					C				
P9 (child aged 13 years)	C	C			C	C						C (Possible restless leg syndrome)

^a^ C—Current sleep problems refer to problems which are currently present, but it should be noted that the duration of these problems is generally longstanding (since infancy or beyond), with most problems also being also present in the past. P—Some parents also reported past sleep problems, which relate to issues that have now been resolved but which were significant problems at some point.

Participants were recruited by responding to online advertisements, which were placed on the websites of epilepsy organizations and charities (e.g., Epilepsy Action), the CASTLE study and researchers' university websites. Participants were recruited between March and July 2018, with interviews taking place between March and July 2018. The criteria for participation were being the parent of a child with epilepsy, based in the United Kingdom and having sufficient English skills to partake in the interview and also willing to review a draft online sleep intervention which was part of the larger CASTLE programme of studies reported in a separate paper.^22^

### 2.2 Measures and data analysis

#### 2.2.1 Interviews

A semi‐structured interview schedule was developed by the researchers that asked about key topics related to the child's sleep and relevant to the development of an online sleep intervention for a wider study (Supporting Information: 1 for full interview schedule; Cook et al.^21^ and Wiggs et al.^22^ for development of the sleep intervention).

#### 2.2.2 Analysis

Data were thematically analysed according to the six standardized stages outlined by Braun and Clarke.^23^ Data familiarization was achieved through the reading and rereading of transcripts before the data were fully coded by a researcher not involved in the interview process (GC). This study employed an inductive analytic strategy driven by the data and participants' own words. Coding was reviewed and discussed amongst the research team, to address any discrepancies and reach agreement. A number of codes were combined following discussions, for example, the codes ‘frequent overnight awakenings’ and ‘night waking issues’ contained similar content and were combined. Other codes that did not relate to mothers' perceptions or experiences around their child's sleep were set aside. During the ‘search for themes’ stage, one of the researchers (GC) reviewed the codes and clustered them into potential themes and subthemes. Next, coded extracts of raw data were revisited, and the themes/subthemes reviewed across the whole data set to ensure that they comprised an accurate reflection of the transcripts. During the ‘defining and naming themes’ stage, the research team reviewed the proposed analysis and refined the specific details of themes, as well as agreed names and descriptions. This involved ongoing discussion and iterative amendments until agreement was reached. The final set of themes and subthemes were agreed upon between all authors.

#### 2.2.3 Procedure

Once parents had expressed an interest (through responding to online adverts), they were provided with an information sheet and given an opportunity to ask any questions. Eligible participants who wished to participate were required to complete a consent form. Interviews were then completed by two researchers (PG and LW) at a time and in a format convenient for participants (two face‐to‐face, six by telephone and one via video call). Interviews were audio recorded, transcribed verbatim and thematically analysed.

## 3 RESULTS

Interviews lasted, on average, 61 min. Themes are presented in line with the study's research questions; four represent the nature of maternally reported sleep problems; seven (and associated subthemes) represent mothers' experiences and feelings around managing their child's sleep and any associated problems and one relates to mothers' perceptions of the available help and support to them around sleep. Themes (and subtheme(s) where appropriate) are presented and described below, with detailed illustrative quotations appearing in Table 3.

**Table 3:** Summary of themes and subthemes supported by detailed quotations

Research question 1: Parentally reported sleep problems faced by CWE and their families	
Theme: Settling issues	‘She had trouble falling asleep, so falling asleep sometimes lasted more than an hour to make her fall asleep’ (P5). ‘He used to just, like, when we put him to bed it would be, like, 2 hours or so afterwards before he'd finally managed to drop off to sleep. Yeah, he really used to struggle to get to sleep and anything like that…’ (P4). ‘If he can't sleep he's up down, annoying his brother, in and out of his sisters room, up and down the stairs … wandering around the house making a noise’ (P6). ‘…it got to the point, even at that age, where you dreaded bedtime with her. You were, like, oh god it's bedtime. It just came over you like a black cloud, your heart sunk, we both looked at each other, like, it's bedtime’ (P7).
Theme: Night‐waking issues	‘He would go to bed fine and go to sleep fine but it's just in the middle of the night when he wakes up … it would be that bit that would cause the problem for us’ (P1). ‘So whether they were seizures waking him up or whether he was just not falling to sleep properly, not going into deep sleep and he was just dozing’ (P2). ‘I'm not sure if they were seizures waking up, because obviously he has quite a lot of seizures. So whether they were seizures waking him up or whether he was just not falling to sleep properly, not going into deep sleep and he was just dozing’ (P2). ‘It's just the amount of times he wakes up and gets up in the night and unless you sit and watch him all night I don't know how you would prevent him getting up all night’ (P6). ‘He'd go to sleep at sort of 8.30/9 and then wake up at between 7 and 8 so he was, but he would always in the night he would wake up and come into us and we'd have to go and put him back’ (P1).
Theme: Parasomnias	‘Well he's 11 this November probably from the age of 7 he's suffered with night terrors intermittently. So we had a few episodes of that and then he did a bit of sleepwalking and he's never since sort of being the age of 7 years old he's never really fully settled at night time’ (P1). ‘But she very much still every night still shouting ‘no, no, no, no' and shouting out … During her sleep yeah, she has a recurrent dream … when she's having the dream and she was shouting “mum, mum” and I'm like “I'm here”’ (P3). ‘Sometimes it was very difficult to tell if he was just wandering around because he didn't want to go to sleep because he was anxious or he'd had a seizure or he was literally sleep walking … he was asleep, so he did sleep walk quite a lot’ (P2).
Theme: Child anxiety around sleep	‘He's always been quite a nervous sleeper you know he's always had to have a light on and things’ (P1). ‘He was having a lot of difficulty sleeping because he was worried that if he went to sleep, because we had quite a few instances where he went to bed as normal and when he woke up he was in hospital. Because sometime in that night he'd had a tonic clonic and we had to call an ambulance because his tonic clonics tend to be about 10 minutes long and his oxygen levels drop, so we have to call an ambulance. So he did go through a stage of not wanting to go to sleep and I think I wrote in there that he had a massive fear that he was going to die in his sleep. So I think that's, it's not just the seizures it's the emotional side of it as well’ (P2).
Research question 2: Parents' experiences and feelings around managing their child's sleep and any associated problems	
Theme: Longstanding challenging nature of child sleep issues	‘I said to him yesterday how's your sleep and he said to be honest I don't think I've slept through the night for the last 6 months’ (P2). ‘He used to just be saying I can't sleep mum I can't sleep all the time and you, it's just awful when they want to sleep but they can't get to sleep’ (P4). ‘It's so draining for her and us really, you know. Because when she's awake she's like Bagpuss the whole house is awake. So she's got a little sister so then she's awake because [child's] awake … It affects everyone’ (P7).
Theme: Management strategies for child sleep	‘He's never slept and we've gone through lots of different things, of early to bed, late to bed, exercise before bed, everything’ (P2).
Subtheme: Co‐sleeping	‘Practical wise it was easier for him to be in with us because he is there all night. Rather than sort of lying there uncomfortably with my arm up in the air trying to hold his hand and then creeping out of the room half an hour later’ (P2). ‘Ever since he had his first seizure he co‐sleeps with his dad because we don't feel comfortable to let him sleep on his own … we'd just be up all night checking on him … it's us more than him’ (P1). ‘But the main thing was we all slept together … I think I was probably 50/50 us wanting to sleep with him as well as him wanting to sleep with us’ (P2). ‘Well he [child] slept in my bed and my husband slept on the floor in our room bless him, on and off for about 3 years’ (P2). ‘…we have to deal what we're living with at the minute and just get on with it and as long as we all get sleep … however we feel, we just feel a lot calmer and more settled if one of us is with him’ (P1).
Subtheme: Sleep environment adaptations	‘It's petrifying isn't it if you wake up and it's pitch black and you're not sure, so he did have various night lights throughout the years, until he was a bit older and now he's got a sort of electric one he can flick on when he wants to by the side of the bed’ (P2). ‘We had one of those safety pillows and I think that's a massive help and it's a massive reassurance for [child] and for parents that you've got one of these safety pillows. That if they do have a tonic clonic face down … they can breathe through the pillow, yes I think that's a massive thing’ (P2). ‘The early nights and everything that we're doing, we've got we've bought black out curtains, we've bought well 2 sets of black out blinds and curtains, we have a fan going, we have all these kind of little interventions that we've put in place just anecdotal’ (P3). ‘Now whether or not that [blackout blinds] cancels out the noise of the rest of the just the general household as well. Because then her brother will be going to bed an hour later. You know there's just those things that I'm very conscious of might wake her up again’ (P3).
Subtheme: Use of monitoring devices	‘He had a couple of tonic clonics in the middle of the night, which we did pick up on because I had this video monitor and you know I had like an alarm and everything … I have that on my phone pointed at a video monitor. So that if he moves on the video monitor even if I don't hear it, it'll pick up the movement as well and sound an alarm, so we did beef up on the seizures’ (P4). ‘Once I got all the monitors setup and everything, I was kind of happy that I wasn't going to miss any [seizures], if he did have any’ (P4). ‘We have monitors as well and I think which have given her some security. I think they help her to go to sleep knowing that she has a monitor … She feels less anxious because she's got a bed monitor and she's got a room like a video monitor on’ (P3). ‘Obviously it's movement [that is monitored by the device] and he's a bit of a rough sleeper so that goes off quite often … I just check on the video monitor and if I can see he's just turning over or doing a normal movement then I know he's fine. If I can't see him properly on the monitor then I'll just pop in and make sure he's alright’ (P4). ‘My problem with the seizure detection monitor is that I've not seen it work, it's not proved itself to me because we've only had it 2 weeks and she hasn't had that kind of seizure’ (P3).
Theme: Challenges related to managing sleep over time	‘It's very hard if she's resistant to want to go bed … because of your seizures. You don't want to bring it into that just before you go to bed. But for an older child they've got to take ownership of it a little bit’ (P4). ‘We have had relaxation DVDs in the past, before she was diagnosed with epilepsy … They were kind of like a story that went into relaxation … We did use those for a good while … but then she outgrew them’ (P3).
Theme: Link between sleep and seizures	‘…a vicious circle because the seizures were caused by lack of sleep and … they're getting stressed because they're not getting enough’ (P4). ‘I know for a fact that if we put him back in his own bed he would be waking up in the night and then obviously we're told that maybe one of the causes of the seizure is lack of sleep and tiredness so we don't want him to, we're scared if he goes back in his own bed and he's waking up all the time is it going to bring on a seizure’ (P1). ‘I was kind of neurotic with trying to make sure that he was getting enough sleep. Because I knew that that was a major trigger for his seizures, because it was when he was tired, he always had more’ (P4).
Theme: Negative impact of poor sleep on daytime functioning	‘Sleep obviously has a massive effect on the way he functions during the day’ (P2). ‘If he's been up then he really does suffer the next day at school, whether that's a knock on effect that he's tired so he has more seizures and its just a constant knock on effect’ (P2). ‘He doesn't do well without sleep and I think that's the other big thing, he does need a lot of sleep and again I don't know whether that's because he wakes numerous times or whether he's a teenager’ (P2).
Theme: Antiseizure medication	‘I mean she still wakes up but since we, I've noticed improvements since we started medicating, that's also the truth’ (P5). ‘We finally found a medicine that stopped the tonic clonics and him just having the partial focals that then I think that that was the turning point that it seemed to be much easier to be much easier to cope with that we knew he wasn't in his bedroom having tonic clonics on his own’ (P2) ‘Because we go to clinic and they say she needs to sleep because you know the better the sleep the less seizures but then they go but she won't sleep because of the condition and the medicine. Side effect is it won't, she won't sleep’ (P7).
Theme: Maternal concerns about child sleep	‘I think it's just that now because at the point of diagnosis they said this is you know the one thing you can do. It feels more pressured’ (P3).
Subtheme: Maternal anxiety around night‐time seizures	‘The fear of it [a seizure] happening and us not hearing him or not being there is just unbearable to think about so for us as a family it's just working [co‐sleeping] at the minute that he's with us’ (P1). ‘It's drilled into you, you need to call an ambulance if the seizure goes on after 5 minutes. If I'm not in her room, if I'm not half awake and alert to when she starts the seizure, how do I know it's gone over 5 and I think that's the main worry for everybody really. So probably for me, the only thing that would make me more comfortable is if I am confident that something would alert me’ (P3). ‘The only thing that would make me more comfortable is if I am confident that something would alert me [to child seizure]’ (P3).
Research question 3: Parents' perception of available help and support when parenting a CWE around sleep	
Theme: Lack of information, help and support available	‘I think even in all the pamphlets and leaflets and whatever we've been given over the years, nobody's ever mentioned sleep’ (P2). ‘Sleep is the one thing that we can do, help but then not really much assistance comes along with that at that point of diagnosis’ (P3). ‘His consultant gives you all sorts of stupid advice and you're like really, “don't let him drink coffee” and I'm like he's 9 I don't let him drink coffee anyway’ (P6). ‘…we are given nothing in hospital, you know or when we've been to A&E, there's nothing that you are told, as I say all we were told at that point is you must get good sleep and so it would be great if somebody then said and here have a look at this … and I would have come straight home and gone straight onto it’ (P3).

Abbreviation: CWE, children with epilepsy.

### 3.1 Research question 1: Parentally reported sleep problems faced by CWE and their families

#### 3.1.1 Settling issues

Mothers commonly reported settling issues, *‘She had trouble falling asleep’* (P5). In many cases, it was the duration in settling their child to sleep which was problematic, such as, *‘2 hours or so afterwards before he'd finally managed to drop off to sleep’* (P4).

In some families, the CWE settling issues negatively influenced the wider family, *‘annoying his brother, in and out of his sister's room…’* (P6). For some, the extent of settling issues influenced their feelings and experiences around putting their child to bed, *‘…you dreaded bedtime with her…’* (P7). Settling issues were problematic for mothers, families and CWE but were one of the most commonly reported sleep problems.

#### 3.1.2 Night‐waking issues

Mothers also commonly reported issues around night wakings, *‘…it's just in the middle of the night when he wakes up…’* (P1). For some mothers, there was ongoing uncertainty around the cause, and some mothers felt helpless about how they could address their child's night waking, *‘…I don't know how you would prevent him getting up all night’* (P6).

These night wakings would also disrupt parents' own sleep, *‘…in the night he would wake up and come into us and we'd have to go and put him back’* (P1). There was an ongoing challenge for parents in dealing with frequent night wakings, perhaps particularly in CWE when the underlying cause of the waking is unknown.

#### 3.1.3 Parasomnias

Nightmares, sleep waking and sleep terrors were common parasomnias experienced by some CWE, *‘…he's suffered with night terrors intermittently … and then he did a bit of sleepwalking…’* (P1). For some mothers, their child's parasomnias were shocking and required parental intervention, *‘when she's having the dream and she was shouting “mum, mum” and I'm like “I'm here”’* (P3). Some mothers specifically highlighted the problems of distinguishing and understanding these behaviours in their CWE. For example, *‘…it was very difficult to tell if he was just wandering around because he didn't want to go to sleep because he was anxious or he'd had a seizure or he was literally sleep walking…’* (P2).

Parasomnias could be longstanding and challenging for parents and children to manage.

#### 3.1.4 Child anxiety around sleep

A difficulty faced by many mothers was their child's sleep‐related worries, concerns or anxieties, *‘He's always been quite a nervous sleeper’* (P1). Some mothers highlighted that the child's epilepsy and the possibility of sleep‐related seizures was a source of anxiety for their child, *‘…he had a massive fear that he was going to die in his sleep’* (P2).

For some mothers, the challenge was managing a calm and relaxing bedtime when child anxiety was most commonly expressed or shared at this time, *‘That's usually when it comes out [child's anxieties or worries] … just when we're doing the bedtime’* (P3).

A key factor in dealing with sleep in CWE was managing the child's worries that could surface around sleep or the anxieties the child had about sleep due to epilepsy.

### 3.2 Research question 2: Parents' experiences and feelings around managing their child's sleep and any associated problems

#### 3.2.1 Longstanding challenging nature of child sleep issues

For many mothers, the sleep problems experienced by their child were longstanding, *‘So when he was diagnosed when he was 7 [now 11] and I think right from the beginning he had problems with sleep’* (P2). Some mothers acknowledged the challenges they felt about supporting their child's sleep problems, *‘It's just awful when they want to sleep but they can't get to sleep’* (P4).

In some cases, it was not just the mothers, but also the children, that acknowledged their disturbed sleep *‘He said to be honest I don't think I've slept through the night for the last 6 months’* (P2). For many mothers, the sleep problems experienced by their CWE negatively impacted the whole family, *‘It's so draining for her and us … It affects everyone’* (P7). Overall, the evidence in this theme suggested that sleep problems were common and longstanding, and their influence was negative for the CWE, their parents and the wider family unit.

#### 3.2.2 Management strategies for child sleep

Many mothers reported adopting certain approaches or practices to facilitate what they perceived to be safe sleep for their children. These were broad and varied in nature and encapsulated within three subthemes.

##### Co‐sleeping

Some mothers found advantages of co‐sleeping (bed and/or room sharing), *‘Practical wise it was easier for him to be in with us because he is there all night’* (P2). For some parents, a key motivation for co‐sleeping was so that they could monitor their child and their safety overnight, which allayed some parental concerns, *‘…we don't feel comfortable to let him sleep on his own’* (P1).

In other cases, co‐sleeping was beneficial as having parents nearby helped to alleviate some of their child's fears or worries, ‘*I think I was probably 50/50 us wanting to sleep with him as well as him wanting to sleep with us’* (P2). However, adopting co‐sleeping practices had a knock‐on impact on the sleep arrangements of the rest of the family, *‘[child] slept in my bed and my husband slept on the floor’* (P2).

For some, co‐sleeping was reactively adopted to facilitate improved familial sleep, *‘…we have to deal with what we're living with at the minute and just get on with it … we just feel a lot calmer and more settled if one of us is with him*’ (P1). Co‐sleeping was a commonly reported strategy desired and adopted by parents and their CWE to provide support and reassurance around child safety.

##### Sleep environment adaptations

Another key strategy was making changes or amendments to their CWE's sleep environment. There were a number of motivations for doing so, for some, it was to make it more comfortable and/or comforting for their child, *‘…he did have various night lights throughout the years’* (P2).

Some parents made use of specific products to provide reassurance to themselves and their child, *‘We had one of those safety pillows and I think that's a massive help and it's a massive reassurance for [child] and for parents’* (P2). In some cases, specific changes were implemented at prominent transitions, for example, when transitioning the CWE to solo sleeping to provide additional assurances.

It was common for mothers to make multiple amendments in an attempt to help manage their child's sleep, even if the evidence base for these specific adaptations was not clear. For example, one family reported, *‘We have all these kind of little interventions that we've put in place, just anecdotal’* (P3).

There was a range of different amendments that mothers made to their child's sleep environment, with the motivations predominantly revolving around offering reassurance and alleviating some safety concerns.

##### Use of monitoring devices

A number of mothers reported using various different types of monitoring devices with their CWE. These allowed parents to monitor their child and/or be alerted to any seizures and intervene if needed. For example, *‘We did pick up on [child's seizures] because I had this video monitor and you know I had like an alarm and everything’* (P4).

The use of monitoring devices was beneficial predominantly due to the reassurance they were perceived to provide; for the mother, *‘Once I got all the monitors setup and everything, I was kind of happy that I wasn't going to miss any [seizures], if he did have any’ (P4) and for the child, ‘We have monitors as well and I think which have given her some security’* (P3).

There was also a range of issues and concerns about the use of these devices. These were around them going off unnecessarily, *‘…loads of false alarms’* (P4) and the alerts could have a disruptive effect on parental sleep because *‘[alarm] goes off quite often’* (P4). In addition, the focus monitors appropriated could be a challenge for mothers who did make use of such devices, *‘Another parent I've heard had a monitor in their room just the video monitor and they said they sat and watched it’* (P3).

Some mothers had reservations about the use of these methods as they did not feel these methods were acceptable for their child, *‘…kind of feels like an invasion of privacy’* (P6). Another concern for some was the reliability and efficacy of monitoring devices, *‘It's not proved itself to me’* (P3).

Monitoring devices were relatively widely used and appeared to offer highly sought‐after reassurance to both parent and child that seizures would not be missed. However, these systems had limitations for some families.

#### 3.2.3 Challenges related to managing sleep over time

The majority of mothers reported that many of the challenges in managing their child's sleep changed over time.

As children became older, they were offered more independence, *‘I can't honestly say whether he was a lot better at sleeping or he was more independent at sorting himself out’* (P2). Mothers also acknowledged that as their child aged and developed, the child needed to take additional responsibility for their epilepsy, *‘…for an older child they've got to take ownership of it a little bit’* (P4).

The type, success and suitability of parental management techniques also evolved over time, *‘We are very conscious that as [child] gets a little bit older she might not want me [to co‐sleep with her]’* (P3). Mothers of CWE clearly highlighted that managing their child's sleep and any sleep problems were challenging, and the type and nature of these challenges tended to change over time.

#### 3.2.4 Link between sleep and seizures

A number of mothers felt their child's sleep and seizures were linked, *‘I know from experience with [child] that if he doesn't get enough sleep then he will have more seizures’* (P2). In some cases, mothers believed that addressing their child's sleep problems would have a positive impact on their seizure management, *‘If I could get sleep sorted I think it would massively change her seizures*. *It's all to do with her sleep’* (P7).

The knowledge of the relationship between sleep and seizures resulted in maternal concerns about ensuring their child obtained enough sleep, *‘I was kind of neurotic with trying to make sure that he was getting enough sleep’* (P4). Mothers were clearly aware of and, in many cases, had experienced first‐hand the relationship between sleep and seizures.

#### 3.2.5 Negative impact of poor sleep on daytime functioning

A number of mothers clearly identified that poor sleep had a negative impact on their child's day‐to‐day functioning, *‘He can be quite sleepy during the day, it depends on what he's doing and obviously if he's seized the night before he's more likely to be tired’* (P6).

A definite link was made by mothers between their child's sleep and potential negative implications for their child's academic endeavours, *‘If he's been up then he really does suffer the next day at school’* (P2). There was a challenge for some mothers in differentiating between poor sleep due to the child's epilepsy or developmental stage, *‘…I don't know whether that's because he wakes numerous times or whether he's a teenager’* (P2). From their own experiences, many mothers linked poor sleep with having negative implications for children's daytime functioning and sleepiness levels, although the cause of poor sleep was not always clear.

#### 3.2.6 Antiseizure medication

A number of mothers reported antiseizure medication having an impact on their child's sleep. For some, antiseizure medications had a positive impact on their child's sleep, *‘I've noticed improvements since we started medicating’* (P5). A benefit of anti‐seizure medication was that it provided additional reassurance for the child, *‘…not kind of worrying about going to sleep like he used to’* (P4).

Another benefit was the additional reassurance this provided that night‐time seizures were not being experienced by the child and so could be missed by parents, ‘We finally found a medicine that stopped the tonic clinics *… it seemed to be much easier to cope with that when we knew he wasn't in his bedroom having tonic clonics on his own’* (P2).

However, for some families, the child's antiseizure medications posed challenges due to their negative impact on child's sleep, *‘…then they go but she won't sleep because of the condition and the medicine’* (P7).

The belief and/or experience that medication‐assisted in reducing and/or assisting in managing seizures gave some CWE and their parent's reassurance around seizures which in turn had a beneficial impact on their child's sleep. However, this positive experience was not shared by all families.

#### 3.2.7 Maternal concerns about child sleep

A number of mothers reported having worries and concerns about sleep for their CWE. For some mothers, even attempting to establish and maintain healthy sleep in their childhood itself was anxiety‐inducing. The fact that sleep is the one aspect highlighted to parents as modifiable added to maternal anxiety about sleep.

##### Maternal anxiety around night‐time seizures

Regardless of the nature of the child's seizures (i.e., frequency and severity), some mothers held concerns about the possibility of night‐time seizures. Particularly noted was the role of the parent in monitoring the child's safety and the impact this had on many families sleeping arrangements, *‘The fear of it [seizure] happening and us not hearing him or not being there is just unbearable to think about’* (P1).

The specific timing of some children's seizures and the challenges of parents monitoring night‐time seizures generated maternal anxieties, *‘If he's getting up in the middle of the night and I'm fast asleep it's much easier to miss if he had a seizure’* (P4). These concerns appeared to be compounded by an awareness of the critical role of parental or medical intervention for child safety.

The timing and safety of children who were likely to experience seizures around sleep or overnight contributed to specific maternal worries and anxieties about sleep.

### 3.3 Research question 3: Parents' perception of available help and support when parenting a CWE around sleep

#### 3.3.1 Lack of information, help and support available

Most mothers had experienced a lack of relevant help and support about sleep issues, *‘We've not had any advice beyond that. Apart from “get good sleep, that's the one thing you can do”’* (P3). This was particularly noted as a salient issue around the time of a new diagnosis.

In some cases, sleep advice that had been offered by healthcare professionals had not been well received or deemed as helpful by mothers. Some even suggested that this lack of information needed to be addressed and suggested the need for standardized advice in the form of a resource that parents could be directed to by healthcare professionals. For example, one mother reported, *‘…it would be great if somebody then said and here have a look at this … I would have come straight home and gone straight onto it’* (P3). It was clear that help and support for child sleep problems were lacking in this clinical group. There is a key need for additional resources or interventions that address sleep in CWE to support parents throughout their epilepsy journey.

## 4 DISCUSSION

Mothers reported four themes which represented the nature of the child's sleep problems. These included settling and night‐waking issues, parasomnias and child anxiety around sleep. Seven themes represented mothers' experiences of managing their child's sleep and any associated problems. These encompassed the longstanding challenging nature of child sleep issues, management strategies adopted, challenges related to managing sleep over time, the link between sleep and seizures, the negative impact of poor sleep on daytime functioning, the role of antiseizure medication and maternal concerns about child sleep. One theme represented the perceived lack of information, help and support available.

The current study contributes to existing empirical evidence that suggests sleeplessness problems and parasomnias are common in CWE^12^, ^14^, ^24^ and that CWE are susceptible to experiencing sleep‐related anxiety around sleep, particularly if they frequently experience seizures or have had traumatic seizure experiences associated with sleep.^25^

There are clear added challenges for parents of CWE when managing their children's sleep and any sleep problems. These concerns commonly resulted in additional maternal monitoring of the child during the night, which is likely to negatively impact both maternal anxiety and sleep, the latter of which is noted to be commonly disrupted in parents of CWE.^14^, ^15^, ^26^, ^27^ It appeared that the use of some management strategies, such as room sharing or co‐sleeping, were often reactively implemented in an attempt to help the CWE and their family obtain the best quantity and quality of sleep that they could, in the circumstances. However, practices such as co‐sleeping can have a detrimental effect on the parent/caregivers' and children's sleep.^14^

In many cases, it appeared that parents employed multiple, sometimes diverse, strategies. A number of mothers reported using different products or devices primarily to help provide some reassurance about their child's safety and security, principally not missing night‐time seizures. There is a wide range of monitoring devices available, but the evidence for their efficacy is scant for children.^28^ Mothers' experiences or concerns around false alarms are also reiterated in the literature in the context of challenges around sensitivity and specificity around these devices.^29^ There is currently no individual seizure‐monitoring device, which is effective for detecting all types of seizures in adults or children.^30^ In addition, a topic of key concern to parents is Sudden Unexpected Death in Epilepsy (SUDEP),^31^ yet no devices have been demonstrated to prevent SUDEP.^32^ The lack of good quality evidence for the use of seizure monitoring devices may make it challenging for parents to select the most appropriate device and to have confidence in its usage.

Mothers were aware of the links between sleep and seizures, yet felt that they lacked guidance about how to address or improve their child's sleep, including from their healthcare teams. This appeared to heighten maternal anxieties and feelings of ‘helplessness’. This finding emphasizes the need to ensure adequate help and support is available to help support healthy sleep in CWE, as identified in previous work.^19^, ^21^ Current results suggest this is crucial around the time of diagnosis and concur with previous research that there is a need for ongoing information and support, beyond diagnosis.^33^ The availability of ongoing, sleep‐related support is an important unmet need for this clinical group.

Some research groups (such as CASTLE) have recognized this gap in sleep‐related help and support of CWE and their families and have begun to explore sleep interventions for this clinical group, the efficacy of which is currently being investigated through a clinical trial (see https://castlestudy.org.uk/). For this group of children, the impact of antiseizure medications on sleep is also a priority, given that antiseizure medication was noted by mothers in the current study to have mixed effects on sleep, as has been reported by others.^34^

The current findings need to be considered in the context of some limitations. The sample size (*n* = 9) was smaller than intended and reflected recruitment challenges. However, it included mothers of a range of diverse participants (child age, epilepsy type and severity and sleep problems), and this heterogeneous group is a distinct strength as this allowed us to obtain a range of parental experiences and perceptions. The original intention had been to recruit both mothers and fathers. However, our final sample solely consisted of mothers. It is not clear if fathers share the same concerns and approaches to managing their child's sleep. In addition, the involvement and availability of the father in co‐habiting households may directly or indirectly influence maternal feelings and behaviours and understanding these dynamics within families needs further elucidation in future work. Importantly, our sample was also self‐selecting and so is unlikely to be representative of all parents of CWE. The current study focused on the parental experience of managing their child's sleep, but it is also important to identify how CWE feel about their sleep and its management. Future work could explore parental and child lived experiences in a larger and more diverse sample. Such information can inform the development of support for families. For example, additional content of the interviews considered in the current study addressed what parents wanted from an online sleep intervention. These have been reported elsewhere,^21^ and some of the key issues highlighted were considered in the development of an online intervention specifically designed for this clinical population.^22^ Addressing sleep in CWE, and its management may have a twofold benefit of improving both the CWE and their parent's sleep quality.

Given all of the authors are experienced sleep and/or epilepsy researchers, while every attempt was made to put aside what we know about sleep and its management in CWE, it is possible that our existing knowledge and understanding (of both the wider literature and conducting qualitative studies with this patient group) influenced the data collection (e.g., following up with questioning on specific sleep issues or problems) and in the analysis of the data (e.g., identifying codes informed by our existing knowledge). However, every attempt was made to foreground parents' words and experiences in the data collection and analysis.

## 5 CONCLUSION

To provide the most appropriate services and support for sleep issues in this patient group, it is essential that the lived experience of parents and the issues and problems that they face in managing their child's sleep are understood and acknowledged. The current paper has highlighted the key areas of child sleep behaviour and associated issues which mothers with lived experience have faced and, in many cases, desire help and support for. Current findings suggest that there are unmet needs in supporting parents to deal with sleep, sleep problems and their management in CWE. Given the link between sleep and seizures, sleep should be an intervention target for health services; if appropriate provision is provided for CWE and their parents, this may result in improved outcomes for multiple child and parental outcomes.

## AUTHOR CONTRIBUTIONS

All authors contributed to the conception and design, acquisition of data, analysis and interpretation of data; and drafting the article or revising it critically for important intellectual content.

## CONFLICT OF INTEREST

The authors declare no conflict of interest.

## ETHICS STATEMENT

Ethical approval was obtained through Oxford Brookes University Research Ethics Committee (study reference 171108). All participants provided written informed consent.

## Supporting information

Supporting information.

## Data Availability

The data sets presented in this article are not readily available because the data are qualitative interviews and cannot be shared. Requests to access the data sets should be directed to amber.collingwood@kcl.ac.uk.
