# Supplementary material for: ‘No one's ever said anything about sleep’: A qualitative investigation into mothers' experiences of sleep in children with epilepsy
Source: Health Expect. 2023 Jan 6;26(2):693–704. doi: 10.1111/hex.13694 (PMC10010080; doi:10.1111/hex.13694)
Supplement: Supplementary file 1 — Supporting information. [file HEX-26--s001.docx]

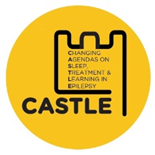
Supplement 1

**Tailoring a behavioural sleep intervention for children with epilepsy: Script for interviews**

1. **General introduction**
   1. thanks for helping us
   2. reminder that session will be audio-taped (content anonymised/treated as confidential), last about an hour or so
   3. reminder that they don’t have to answer any questions they don’t want to
   4. reminder that all views are valid (feel free to speak freely)
   5. reminder that they can withdraw from the study/leave at any time but that any data submitted to that point (via discussion) cannot be withdraw
   6. any questions?
2. **Fill in any missing details below:**

Name of parent:____________________________________________________________________________

Relationship to child: Mother/Father/Other (please describe) ________________________

Telephone number: ________________________________________________________

If we do need to try and speak to you by phone are there particularly good times for us to call? ___________________________________________________________________

Email address):____________________________________

Child’s name:___________________________________________________________ BOY / GIRL

Child’s age:_______________

Does (child) have epilepsy? YES/NO

Type of epilepsy? ___________________________________________________________________

Because in the next phase of this project we will ask you to look at the draft online intervention we develop will you have access to the internet. Is that possible for you? YES/NO

And similarly, just need to check that you are happy that your English of a level that you would find it possible to read the information in any online material? YES/NO

Has your child suffered from sleep disturbances currently or in the past? YES/NO

If yes, what type of sleep problems had they/do they have?

__________________________________________________________________________________

1. **Facilitate discussion around the following areas:**

**What types of sleep problems experienced?**

Are there particular difficulties with sleep faced by the family/child because of the child’s epilepsy? What are the areas related to the child’s sleep that parents want help with?

**What types of sleep interventions have been tried?**

What interventions have been suggested/tried? Success of these? Why lack of success? What things were helpful? Why? What things were not helpful? Why? Parents general feelings about suitability/rationale of behavioural techniques for their child/family? What would help make them more useful/suitable?

**Any particular problems with using behavioural techniques?**

Were there any particular behavioural techniques previously suggested to the family that were difficult to implement or parents did not want to implement? Why? What were the barriers to using these techniques? What could have helped/encouraged them to use a technique? Which techniques were easy to implement?

**Are there any specific issues which are important for parents when dealing with sleep in a child with epilepsy which haven’t been mentioned?**

**What makes a good/bad online (sleep) information seeking experience?**

Length/time taken; look of it (font, colours, amount of information per page); use of videos (parents, clinicians, researchers?); format/content of case studies; use of printed materials; length/use of quizzes; thoughts about using diaries online; ways to navigate through pages (i.e. access to all material or only material specific to your child’s problems; ways to make it seem personal; how to instil confidence in parents; balance of research/practical information

1. Confirm what’s happening next (e.g. reminder of the project flow (*i.e. we will develop intervention based on what parents say then send you details, by email, of how you can log on and look at the draft website*), when the draft version will be available (*mid/end sept*), what they are expected to do with the draft version (*we will send detailed instructions but basically we want you to look at it and see if it’s easy to navigate around the site, whether they like the look of it, whether the information is clear and suitable, how we will contact them etc. – then we’ll have another chat so that you can tell us what you thought*)
2. Reminder of how to claim travel expenses (if face to face)
3. Questions, thank you and goodbye
